# Effects of High Glucose on Simulated Ischemia/Reperfusion Injury in Isolated Cardiomyocytes

**DOI:** 10.3390/ijms26136050

**Published:** 2025-06-24

**Authors:** Miriam J. K. Walter, Masakazu Shiota, Zhu Li, Matthew B. Barajas, Takuro Oyama, Matthias L. Riess

**Affiliations:** 1 Department of Anesthesiology, Vanderbilt University Medical Center, Nashville, TN 37232, USA; miriam_walter@t-online.de (M.J.K.W.);; 2 Department of Anesthesiology, Universitätsmedizin Greifswald, 17475 Greifswald, Germany; 3 Department of Molecular Physiology and Biophysics, Vanderbilt University School of Medicine, Nashville, TN 37232, USA; 4 Department of Anesthesiology, TVHS VA Medical Center, Nashville, TN 37212, USA; 5 Department of Pharmacology, Vanderbilt University, Nashville, TN 37232, USA

**Keywords:** aldose reductase, cardioprotection, diabetes mellitus, diabetic complications, hyperglycemia, reactive oxygen species

## Abstract

The rising prevalence of type 2 diabetes is linked to an increased risk of cardiovascular diseases, with the diabetic heart being particularly vulnerable to ischemia–reperfusion (IR) injury. Chronic hyperglycemia contributes to an increase in reactive oxygen species and impacts the homeostasis of biochemical pathways, including the polyol pathway, increasing susceptibility to damage. Aldose reductase (AR), a key enzyme in this pathway, has been targeted for therapeutic intervention, with AR inhibitors showing potential in mitigating diabetic complications. This study investigated IR injury in cardiomyocytes following high glucose exposure and assessed the AR inhibitor Epalrestat as a protective agent. Cardiomyocyte function was evaluated by measuring lactate dehydrogenase (LDH) release, FM1-43 membrane incorporation, cell viability, intracellular calcium accumulation, and superoxide anion formation. High glucose exposure and simulated IR led to increased LDH release, FM1-43 incorporation, intracellular calcium, and superoxide levels, alongside reduced cell viability in a dose-dependent manner. However, Epalrestat treatment during high glucose exposure significantly reduced IR-induced injury. These findings suggest that high glucose exacerbates IR injury in cardiomyocytes, with the polyol pathway playing a critical role. Targeting this pathway with AR inhibitors like Epalrestat may offer a protective strategy against diabetic heart complications.

## 1. Introduction

Cardiovascular diseases (CVDs) are the leading cause of death and a major cause of disability, with an estimated 20.5 million deaths in 2021 worldwide [1]. Most CVDs are thought to be preventable by modifying lifestyle factors, such as tobacco use, poor nutrition, obesity, physical inactivity, and excessive alcohol use [2].

Myocardial infarction (MI), stroke, and peripheral vascular disease are prototypic illnesses characterized by ischemia/reperfusion (IR) injury. Ischemia is defined as inadequate blood flow to the tissue and, therefore, a deficiency of oxygen and nutrients. Additionally, the lack of blood flow results in an accumulation of potentially damaging by-products [3]. Reperfusion is needed to restore blood flow and oxygen levels. However, before cells can stabilize, reperfusion results in additional injury, known as reperfusion injury [4,5]. The pathophysiology of IR injury is thought to be multifactorial: formation of reactive oxygen species (ROS), calcium overload, endoplasmic reticulum stress, mitochondrial dysfunction, apoptosis, protein kinase activation, and inflammation all play a major role in the IR injury process [3,6,7].

Individuals with type 2 diabetes mellitus (T2DM) without history of myocardial infarction have an equal risk of MI as compared to non-diabetic people with prior MI [8]. The global prevalence of T2DM and the attributable risk for CVDs due to T2DM are constantly increasing [9,10]. Additionally, the diabetic heart and vascular system appear to be more susceptible to IR injury, potentially through disruption of intracellular signaling responsible for increased resistance to cell death, leading to larger myocardial infarct size after reperfusion in diabetic patients compared to non-diabetic patients [11,12,13,14]. An ex vivo heart model showed that, while overall myocardial function was not worse in diabetic compared to non-diabetic animals, infarct size was significantly larger in diabetic animals. An adjustment of glucose levels did not affect infarct size in diabetic animals [15]. The underlying mechanisms responsible for the aggravation of IR injury in the diabetic heart are still not clearly known [11].

**Figure 1:**
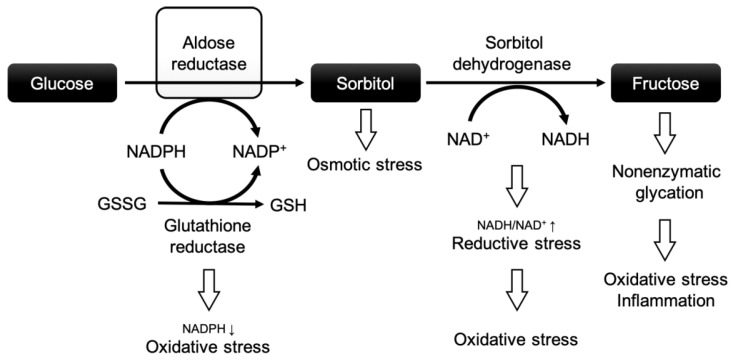
The polyol pathway and the role of aldose reductase in diabetic complications. Modified after Grewal et al. [18]. Abbreviations: NADPH: nicotinamide adenine dinucleotide phosphate + hydrogen; NADP+: nicotinamide adenine dinucleotide phosphate; NADH: nicotinamide adenine dinucleotide + hydrogen; NAD+: nicotinamide adenine dinucleotide; GSSG: oxidized glutathione; GSH: reduced glutathione.

Chronic hyperglycemia contributes to insulin resistance, reduced insulin secretion, and, subsequently, inadequate downregulation of non-insulin-dependent transporters [16,17]. Chronic hyperglycemia not only leads to an increase in ROS but also to a biochemical imbalance, impacting a variety of crucial biochemical pathways, such as the polyol pathway (Figure 1).

In this pathway, a high glucose flux leads to increased turnover via aldose reductase (AR), the rate-limiting enzyme of the polyol pathway. The polyol pathway consists of two reactions: (1) AR reduces glucose to sorbitol by oxidizing nicotinamide adenine dinucleotide phosphate (NADPH), and (2) sorbitol dehydrogenase converts sorbitol to fructose at the expense of nicotinamide adenine dinucleotide (NAD+) [19]. NADPH is an important co-factor for the reduction of glutathione, which protects cells against oxidative injury by neutralizing ROS [20]. In a nutshell, chronic hyperglycemia may negatively affect cardiomyocytes through the polyol pathway by (i) excessive depletion of NADPH (increasing the vulnerability to oxidative stress), (ii) osmotic stress due to intracellular accumulation of sorbitol, (iii) enhanced oxidative stress through the depletion of NAD+ and the production of nicotinamide adenine dinucleotide + hydrogen (NADH) [21,22], and (iv) the excessive formation of advanced glycation end products (further causing oxidative stress and inflammation) [23,24]. These factors are believed to be crucial for a higher vulnerability to IR injury in the diabetic heart and vascular system.

In clinical practice, several therapeutic strategies are employed to mitigate IR injury in diabetic patients: Tight glycemic control, particularly with agents like metformin, has been shown to reduce oxidative stress and cellular injury during IR [25]. Antiplatelet therapies, such as aspirin and P2Y12 inhibitors, are also used to reduce the cardiovascular risk, which is elevated in diabetes and worsens IR outcomes [26]. Moreover, emerging cardioprotective agents, including SGLT2 inhibitors and GLP-1 receptor agonists, have demonstrated beneficial effects in reducing cardiac IR injury during acute myocardial infarction through mechanisms that reduce myocardial infarct size and improve cardiac function and cardiovascular prognosis [27].

**Figure 2:**
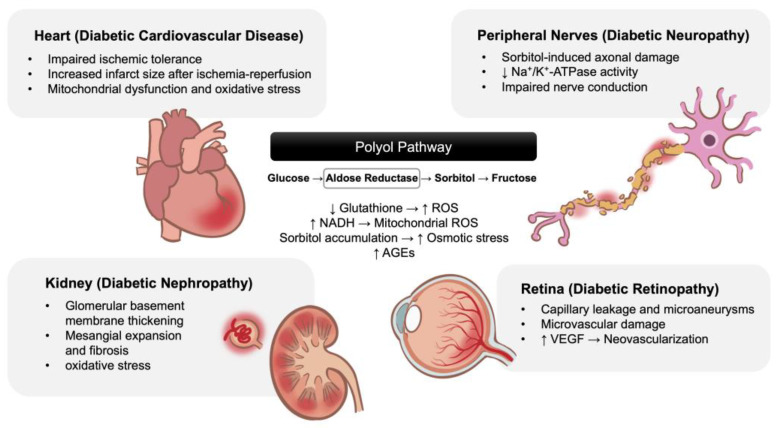
The polyol pathway as a central mechanism linking chronic hyperglycemia to diabetic complications across multiple organs. Aldose reductase activation leads to sorbitol accumulation and increased oxidative stress, contributing to tissue injury in the heart, peripheral nerves, kidney, and retina. Adapted and modified from [28,29,30,31,32]. Abbreviations: ROS: reactive oxygen species; NADH: nicotinamide adenine dinucleotide + hydrogen; AGEs: advanced glycation end products; VEGF: vascular endothelial growth factor.

Despite these advances, treatment of IR injury in the context of diabetes remains a clinical challenge. Diabetic hearts often show impaired responsiveness to conventional cardioprotective interventions, underscoring the need for different therapy approaches that specifically target diabetes-related metabolic dysfunctions [11]. One such promising class includes AR inhibitors as a treatment of diabetic complications (Figure 2).

Among these, several AR inhibitors have shown potential in counteracting glucose-induced oxidative stress and tissue damage, including in the context of IR injury.

Epalrestat has demonstrated efficacy in improving nerve conduction velocity, reducing inflammatory markers, and alleviating symptoms of diabetic peripheral neuropathy when used in combination with mecobalamin (vs. mecobalamin-only treatment) and without increasing adverse effects [33]. It is suggested that Epalrestat helps in the upregulation of nerve growth factor and, consequently, improves the function of peripheral nerves [34]. Furthermore, Epalrestat has been available for the treatment of diabetic retinopathy in Japan since 1992. It is also being investigated as a protective agent in animal models of diabetic nephropathy [35,36,37]. Other AR inhibitors have shown similar promise in preclinical studies.

Cemtirestat has demonstrated protective effects in rat models of diabetic ocular damage by mitigating glucotoxicity-induced tissue injury [38].

Fidarestat was able to reduce the intracellular superoxide anion formation induced by diabetes and protected against cardiomyocyte dysfunction in an in vitro mouse cardiomyocyte model [39].

Similarly, Sorbinil has been reported to prevent retinal microangiopathies in diabetic rats [40].

These findings underscore the therapeutic potential of AR inhibitors in reducing diabetes-related oxidative stress and tissue injury, including in cardiovascular contexts. Although their clinical application outside of neuropathy and retinopathy remains limited and under investigation.

Given the growing burden of diabetes and the high cardiovascular risk in this population, a better understanding of the mechanisms by which hyperglycemia exacerbates IR injury is essential. Despite favorable evidence in other diabetic complications, the role of AR inhibition in protecting cardiomyocytes from IR injury under high-glucose conditions remains largely unexplored. To our knowledge, this is the first study assessing an AR inhibitor in an in vitro model of hyperglycemia combined with simulated IR injury. In vitro models allow for a more controlled environment and offer higher reproducibility and accessibility compared to in vivo models.

In brief, this study aimed to:

- Investigate the injury process in cardiomyocytes after exposure to high glucose concentrations followed by simulated IR injury.
- Assess the potential of the AR inhibitor Epalrestat to attenuate simulated IR injury after high glucose exposure.

## 2. Results

### 2.1. Effects of High Glucose Exposure Followed by Simulated Ischemia/Reperfusion Injury in Cardiomyocytes

**Figure 3:**
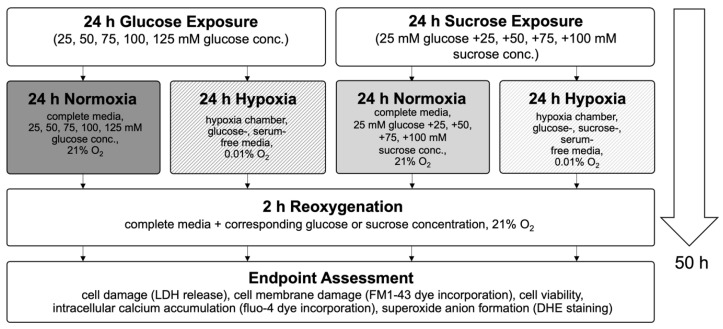
Flow chart of experimental timeline of the high glucose exposure hypoxia/reoxygenation injury model. Abbreviations: LDH: lactate dehydrogenase; DHE: dihydroethidium.

In this study, we used an in vitro hypoxia/reoxygenation (HR) model to simulate ischemia/reperfusion (IR) injury in H9c2 cardiomyoblasts. Cells were exposed to increasing concentrations of glucose (25–125 mM) or to iso-osmolar sucrose controls (containing 25 mM glucose) to evaluate the impact of hyperglycemic and hyperosmolar stress. After 24 h of treatment, the cells were subjected to either 24 h of hypoxic conditions (to mimic ischemia) or maintained under normoxia. Reoxygenation (simulated reperfusion) was performed for two hours, followed by endpoint assessments, including cell viability, LDH release, membrane integrity (FM1-43 dye incorporation into the cell membrane), intracellular calcium accumulation, and ROS generation (Figure 3). Details on normalization and statistical testing, a detailed description of the experimental design, and assays can be found in Section 4: Materials and Methods.

#### 2.1.1. Cell Damage Assessed by Lactate Dehydrogenase Release

**Figure 4:**
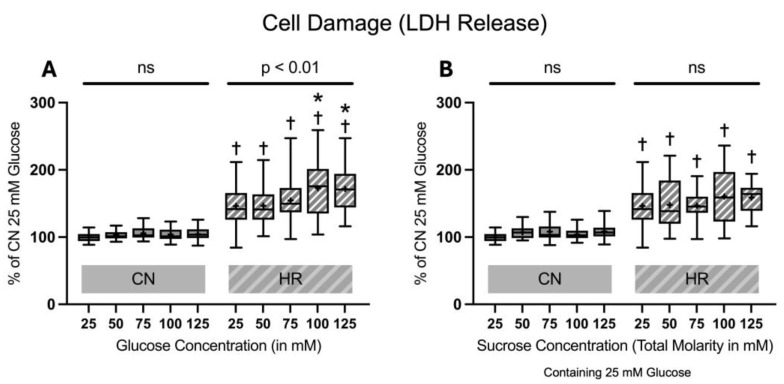
Lactate dehydrogenase (LDH) release after 24 h of (**A**) glucose or (**B**) sucrose exposure, 24 h of hypoxia and 2 h of reoxygenation (HR), or control/normoxic (CN) conditions; n = between 28 and 32 per group. All results are normalized to the control group with 25 mM glucose under normoxic conditions (CN 25 mM), which was set to 100%. Data are presented as boxplots (mean indicated by “+”; boxes: 25th–75th percentile; whiskers: min–max). Statistical significance was assessed using ANOVA or Kruskal–Wallis tests as appropriate, with *p* < 0.05 considered significant. Symbols: † *p* < 0.0001 vs. CN 25 mM glucose; ns = not significant; * *p* < 0.05 comparison of glucose/sucrose concentration group after HR vs. HR 25 mM glucose. Comparisons not labeled were not statistically significant. Note: CN 25 mM glucose and HR 25 mM glucose groups are identical in panels (**A**,**B**), as they were derived from the same wells on the same plate and are shown in both panels for clarity.

After 24 h of glucose or sucrose exposure and a subsequent 24 h of hypoxia with two hours of reoxygenation, there was a strong increase in LDH release between the HR as compared to the CN groups (46.0% LDH increase between cells exposed to 25 mM glucose and HR and cells exposed to 25 mM glucose and CN conditions). Increasing glucose concentrations led to an additional increase in the LDH release under HR conditions in a dose-dependent manner; adding sucrose showed a trend towards increased LDH release but did not reach significance. In contrast, there was no significant increase in the LDH release under CN conditions with increasing glucose or sucrose concentrations (Figure 4).

#### 2.1.2. Cell Membrane Damage Assessed by FM1-43 Incorporation into the Cell Membrane

**Figure 5:**
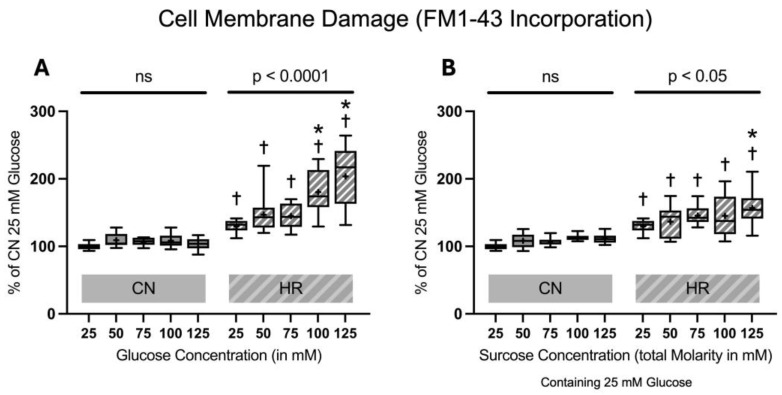
FM1-43 incorporation into the cell membrane after 24 h of (**A**) glucose or (**B**) sucrose exposure, 24 h of hypoxia and 2 h of reoxygenation (HR) or control/normoxic (CN) conditions; n = between 11 and 12 per group. All results are normalized to the control group with 25 mM glucose under normoxic conditions (CN 25 mM), which was set to 100%. Data are presented as boxplots (mean indicated by “+”; boxes: 25th–75th percentile; whiskers: min–max). Statistical significance was assessed using ANOVA or Kruskal–Wallis tests as appropriate, with *p* < 0.05 considered significant. Symbols: † *p* < 0.01 vs. CN 25 mM glucose; ns = not significant; * *p* < 0.05 comparison of glucose/sucrose concentration group after HR vs. HR 25 mM glucose. Comparisons not labeled were not statistically significant. Note: CN 25 mM glucose and HR 25 mM glucose groups are identical in panels (**A**,**B**), as they were derived from the same wells on the same plate and are shown in both panels for clarity.

FM1-43 dye incorporation was significantly elevated in HR as compared to the CN groups (30.0% increase between cells exposed to 25 mM glucose and HR versus cells exposed to 25 mM glucose and CN conditions). Increasing glucose concentrations led to an increase in FM1-43 dye incorporation under HR conditions in a dose-dependent manner. The addition of sucrose also increased FM1-43 dye incorporation at the highest molarity. In contrast, there was no significant increase in FM1-43 dye incorporation in CN conditions with increasing glucose or sucrose concentrations (Figure 5).

#### 2.1.3. Cell Viability and Toxicity

**Figure 6:**
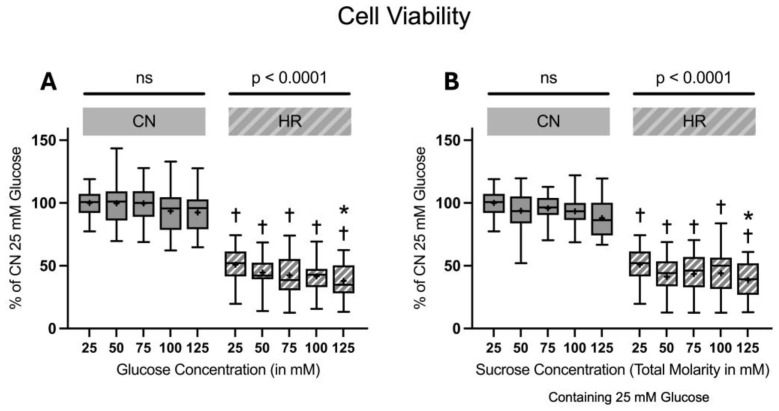
Cell viability after 24 h of (**A**) glucose or (**B**) sucrose exposure, 24 h of hypoxia and 2 h of reoxygenation (HR), or control/normoxic (CN) conditions; n = between 28 and 32 per group. All results are normalized to the control group with 25 mM glucose under normoxic conditions (CN 25 mM), which was set to 100%. Data are presented as boxplots (mean indicated by “+”; boxes: 25th–75th percentile; whiskers: min–max). Statistical significance was assessed using ANOVA or Kruskal–Wallis tests as appropriate, with *p* < 0.05 considered significant. Symbols: † *p* < 0.0001 vs. CN 25 mM glucose; ns = not significant; * *p* < 0.05 comparison of glucose/sucrose concentration group after HR vs. HR 25 mM glucose. Comparisons not labeled were not statistically significant. Note: CN 25 mM glucose and HR 25 mM glucose groups are identical in panels (**A**,**B**), as they were derived from the same wells on the same plate and are shown in both panels for clarity.

The cell viability significantly decreased in HR compared to CN groups (49.1% decrease between cells exposed to 25 mM glucose and HR versus cells exposed to 25 mM glucose and CN conditions). Increasing glucose or sucrose concentrations did lead to a significant decrease in the cell viability in HR cells at the highest molarity. There was no significant decrease in the cell viability under CN conditions with increasing glucose or sucrose concentrations (Figure 6).

#### 2.1.4. Intracellular Calcium Accumulation

**Figure 7:**
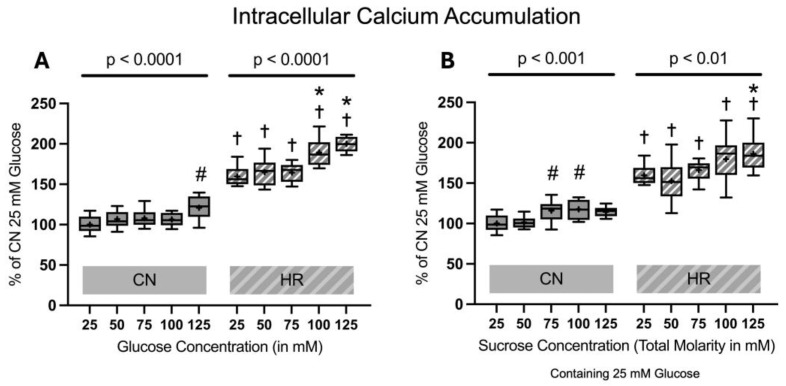
Intracellular calcium accumulation after 24 h of (**A**) glucose or (**B**) sucrose exposure, 24 h of hypoxia and 2 h of reoxygenation (HR), or control/normoxic (CN) conditions; n = between 11 and 12 per group. All results are normalized to the control group with 25 mM glucose under normoxic conditions (CN 25 mM), which was set to 100%. Data are presented as boxplots (mean indicated by “+”; boxes: 25th–75th percentile; whiskers: min–max). Statistical significance was assessed using ANOVA or Kruskal–Wallis tests as appropriate, with *p* < 0.05 considered significant. Symbols: † *p* < 0.01 vs. CN 25 mM glucose; ns = not significant; * *p* < 0.05 comparison of glucose/sucrose concentration group after HR vs. HR 25 mM glucose; # *p* < 0.05 comparison of glucose/sucrose concentration group after CN vs. CN 25 mM glucose. Comparisons not labeled were not statistically significant. Note: CN 25 mM glucose and HR 25 mM glucose groups are identical in panels (**A**,**B**), as they were derived from the same wells on the same plate and are shown in both panels for clarity.

There was a substantial rise in intracellular calcium accumulation in the HR groups as compared to the CN groups (59.6% increase between cells exposed to 25 mM glucose and HR versus cells exposed to 25 mM glucose and CN conditions). Increasing glucose concentrations led to an additional increase in intracellular calcium accumulation in HR cells in a dose-dependent manner. The addition of sucrose also increased the intracellular calcium accumulation, with a significant increase between 25 mM glucose + 100 mM sucrose vs. 25 mM glucose after HR. There was a significant increase in intracellular calcium accumulation under CN conditions, with increasing glucose or sucrose concentrations at higher concentrations (Figure 7).

#### 2.1.5. Superoxide Anion Formation

**Figure 8:**
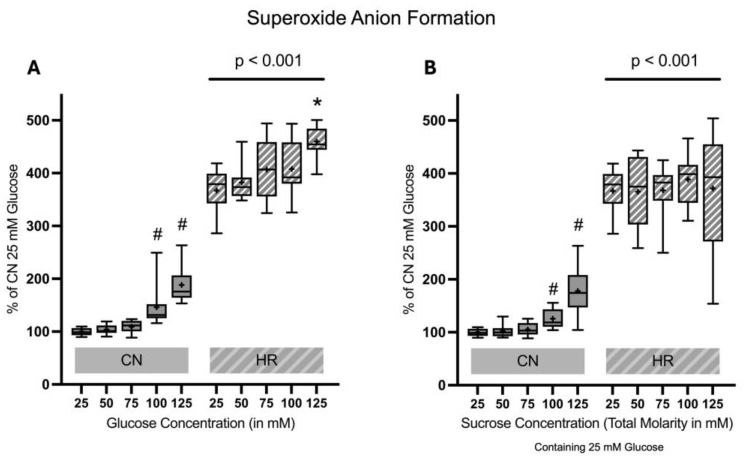
Superoxide anion formation after 24 h of (**A**) glucose or (**B**) sucrose exposure, 24 h of hypoxia and 2 h of reoxygenation (HR), or control/normoxic (CN) conditions; n = 11 to 12 per group. All results are normalized to the control group with 25 mM glucose under normoxic conditions (CN 25 mM), which was set to 100%. Data are presented as boxplots (mean indicated by “+”; boxes: 25th–75th percentile; whiskers: min–max). Statistical significance was assessed using ANOVA or Kruskal–Wallis tests as appropriate, with *p* < 0.05 considered significant. Symbols: ns = not significant; * *p* < 0.05 comparison of glucose/sucrose concentration group after HR vs. HR 25 mM glucose; # *p* < 0.05 comparison of glucose/sucrose concentration group after CN vs. CN 25 mM glucose. Comparisons not labeled were not statistically significant. Note: CN 25 mM glucose and HR 25 mM glucose groups are identical in panels (**A**,**B**), as they were derived from the same wells on the same plate and are shown in both panels for clarity.

Superoxide anion formation was substantially elevated after HR as compared to the CN groups (267% increase between cells exposed to 25 mM glucose and HR versus cells exposed to 25 mM glucose and CN conditions). Increasing glucose but not sucrose concentrations led to an increase in superoxide anion formation in HR cells in a dose-dependent manner. Additionally, there was also an increase in superoxide anion formation under CN conditions with increasing glucose and sucrose concentrations (Figure 8).

### 2.2. Effects of Aldose Reductase Inhibitor on Lactate Dehydrogenase Release of Cardiomyocytes Exposed to High Glucose or Sucrose Concentrations Followed by Hypoxia/Reoxygenation

**Figure 9:**
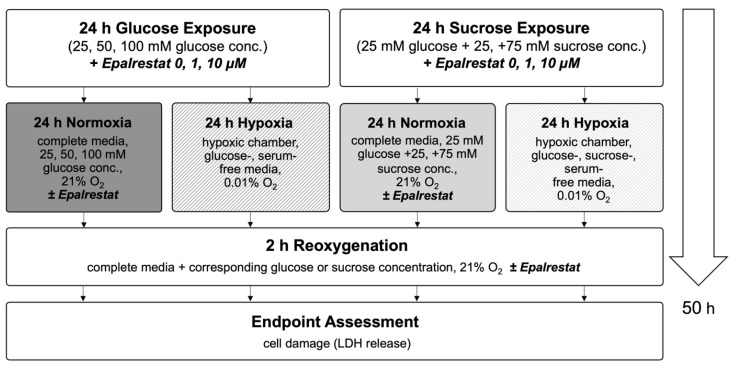
Flow chart of experimental timeline of treatment with the aldose reductase inhibitor Epalrestat. Abbreviations: LDH: lactate dehydrogenase.

To investigate if inhibition of the polyol pathway can mitigate cell injury under hyperglycemic conditions, Epalrestat was added at final concentrations of 0, 1, or 10 µM during the glucose/sucrose exposure period. LDH release was measured following 24 h of glucose/sucrose exposure, 24 h of hypoxia, and two hours of reoxygenation (Figure 9).

**Figure 10:**
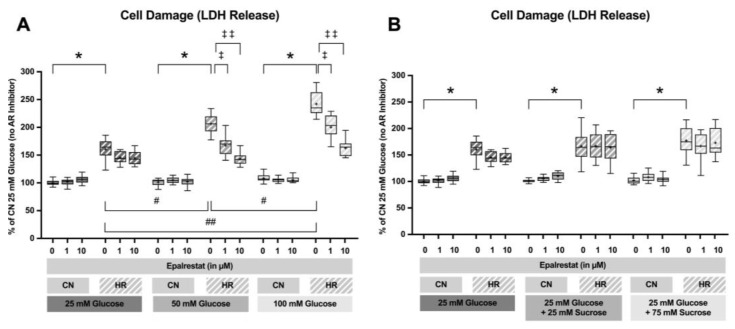
Lactate dehydrogenase (LDH) release after 24 h of (**A**) glucose or (**B**) sucrose exposure and aldose reductase inhibitor (Epalrestat) treatment followed by 24 h of hypoxia and 2 h of reoxygenation (HR) or control/normoxic (CN) conditions; n = between 17 and 18 per group. Data were normalized to the control group with 25 mM glucose under normoxic conditions (CN 25 mM), set to 100%. Data are presented as boxplots (mean: “+”; boxes: 25th–75th percentile; whiskers: min–max). Statistical comparisons were made using ANOVA or Kruskal–Wallis tests, depending on distribution; *p* < 0.05 was considered significant. Symbols: * *p* < 0.0001 HR vs. CN for all groups; # *p* < 0.01, ## *p* < 0.0001 comparison between different glucose/sucrose concentrations; ‡ *p* < 0.01, ‡‡ *p* < 0.0001 Epalrestat (AR inhibitor) vs. no treatment. Comparisons not shown were not statistically significant. Note: CN 25 mM glucose and HR 25 mM glucose treated with 0, 1, and 10 µM Epalrestat groups are identical in panels (**A**,**B**), as they were derived from the same wells and plates (during the same experiments containing all glucose and sucrose groups) and are shown in both panels for clarity.

Adding Epalrestat during the glucose exposure period reduced LDH release after HR by 39.2% and 63.1% at 1 and 10 µM during 50 mM glucose exposure and by 41.7% and 79.5% at 1 and 10 µM during 100 mM glucose exposure. The addition of sucrose did not increase cell injury. The AR inhibitor did not affect cells exposed to sucrose (Figure 10).

## 3. Discussion

Various in vitro hyperglycemia-IR-injury models have been used to test different cell-protective agents in cardiomyocytes [41,42,43,44,45,46]. Yet, the pathophysiological mechanisms that increase the susceptibility of cardiomyocytes to IR injury after hyperglycemic conditions are still a matter of debate. Furthermore, there are currently no studies known to us investigating an AR inhibitor in an in vitro hyperglycemia-IR-injury model.

In this current work, different physiological markers of cell damage were measured: LDH release, incorporation of FM1-43 into the cell membrane, cell viability, intracellular calcium accumulation, and formation of superoxide anion. In comparison to the “CN 25 mM Glucose” groups, cardiomyocytes exposed to different levels of glucose and subsequent HR (simulated IR) showed dose-dependent cell damage based on all the above-mentioned markers. In other words, cell damage was consistently shown across different methodological approaches and with different administered assays.

Notably, under CN conditions, both glucose and sucrose appeared to enhance superoxide anion formation in a concentration-dependent manner. This may reflect the effects of hyperosmolarity, as high extracellular sugar concentrations (regardless of metabolic activity) can induce mild cellular stress, contributing to ROS generation. However, under HR conditions, glucose led to higher increases in superoxide production than sucrose. This suggests that intracellular metabolism of glucose may play a critical role in ROS generation during IR stress. Unlike glucose, sucrose is not readily transported into cardiomyocytes and therefore does not engage metabolic pathways such as glycolysis, the polyol pathway, or mitochondrial respiration, which are key contributors to oxidative stress under HR.

In this study, although not always significant, sucrose treatment also showed a trend towards increased cellular damage after HR but less so than the groups exposed to high glucose concentrations. This suggests that osmolarity, at least in part, might contribute to cellular injury. Adding the AR inhibitor Epalrestat during high glucose exposure reduced the LDH release after HR. In contrast, the AR inhibitor did not affect cells exposed to sucrose. AR is an aldo-keto reductase with substrate specificity for aldo-sugars such as glucose and galactose [47]. Sucrose is a complex disaccharide and, therefore, cannot be utilized by AR.

In the work presented here, no significant difference in the decrease in cell viability after HR between the glucose and sucrose groups was identified. Additionally, increasing sucrose concentrations did not affect LDH release or FM1-43 incorporation after HR. However, changes were observed in cell viability, intracellular calcium accumulation, and superoxide anion formation. These findings can be attributed to the distinct biochemical effects of glucose and sucrose on cardiomyocytes. Glucose, as a metabolizable sugar, participates in metabolic pathways and, therefore, increases oxidative stress, causes mitochondrial dysfunction, and disrupts calcium regulation. On the other hand, sucrose cannot be broken down by cardiomyocytes into its monosaccharides, glucose and fructose. It does not enter metabolic pathways. Thus, sucrose primarily increases osmotic stress without directly affecting cellular metabolism. LDH is released when the cell membrane is damaged, typically as a result of necrosis or late-stage apoptosis. In this study, the damage induced by glucose likely stems from its metabolic effects (e.g., increased oxidative stress through ROS and the polyol pathway), whereas sucrose does not directly damage the membrane but may induce less severe damage through osmotic stress. Hence, LDH release was not affected as much by sucrose as by glucose, as it is primarily a marker of membrane disruption rather than osmotic imbalance. Similarly, FM1-43 incorporation into the cell membrane is a marker of membrane permeability. The absence of increased FM1-43 incorporation with higher sucrose concentrations suggests that sucrose does not cause significant membrane disruption or damage, unlike glucose, which induces membrane injury through oxidative stress and metabolic dysregulation. While osmotic stress can reduce the cell viability by altering the water content, disrupting ion homeostasis, or stressing intracellular structures, it does not necessarily lead to immediate membrane rupture. Cells might die from internal stressors and undergo programmed cell death without the catastrophic membrane damage seen in necrosis [48]. Furthermore, osmotic stress can trigger specific signaling pathways that activate necroptosis, further demonstrating that cell death can occur under osmotic conditions without the need for membrane rupture [49]. The increase in intracellular calcium accumulation and superoxide anion formation at higher sucrose concentrations likely reflects the fact that both processes are more sensitive to osmotic stress. Although sucrose does not directly engage with metabolic pathways, its osmotic effects can disrupt ion homeostasis, leading to an influx of calcium that contributes to cell injury and dysfunction [50]. Whereas glucose induces superoxide anion production through its metabolic impact, osmotic stress from sucrose may also trigger oxidative stress by indirectly affecting ion channels, leading to increased ROS production, albeit to a lesser extent than glucose [51].

### 3.1. Effects of High Glucose and Ischemia/Reperfusion Injury in Cardiomyocytes

Chronic hyperglycemia has been linked to multiple complications (e.g., microvascular complications, including diabetic retinopathy, nephropathy, and neuropathy) [16,28,29,30,31,32,52]. Additionally, it has been associated with macrovascular disease as well as pathophysiologic features of T2DM itself (e.g., basement membrane thickening and formation of advanced glycations end products) [16,53,54,55]. Chronic hyperglycemia leads to decreased insulin secretion and increased insulin resistance, a concept commonly referred to as glucose toxicity [16,54,56]. Moreover, the negative effects of high blood glucose levels may also affect critically ill patients who often show stress-induced hyperglycemia [57,58].

The neuroendocrine system responds to stress by secretion of anti-insulin hormones (glucagon, adrenaline, cortisol, and growth hormone), resulting in increased insulin resistance and decreased insulin secretion with enhanced glycogenosis in the liver. Diabetic patients are more vulnerable to stress, leading to higher incidences of complications and exacerbation of diabetic symptoms [11,57]. The increased oxidative stress in T2DM may result from an imbalance of increased ROS generation and decreased neutralization of ROS by antioxidant defense systems [59]. On the molecular level, chronic hyperglycemia leads to the formation of advanced glycation end products and an altered polyol pathway activity, resulting in inflammatory responses and increased basal oxidative stress [60]. Additionally, the superoxide anion production via the mitochondrial electron transport chain is markedly increased in hyperglycemic conditions [61]. Furthermore, ischemic pre- and post-conditioning (i.e., mechanisms of protection of the ischemic heart) may be less effective in T2DM due to disrupted intracellular signaling and decreased cellular resistance [62,63]. Taken together, this emphasizes the role of IR injury in the diabetic population and warrants further exploration of pathophysiological mechanisms as well as preventive and therapeutic strategies.

### 3.2. Aldose Reductase Inhibitors and Their Cardioprotective Effects

Studies have investigated AR, the rate-limiting enzyme of the polyol pathway, as a potential target for the treatment of diabetic complications [33,34,35,36,37,38,39,40,64,65,66,67,68]. Focusing on the cardiovascular system, the inhibition of AR activity preserved high-energy phosphates, maintained a lower cytosolic NADH/NAD+ ratio, and protected both diabetic and non-diabetic hearts during IR in an isolated perfused heart (Langendorff) model [69].

Administration of Zopolrestat in an isolated perfused heart model found that inhibition of the polyol pathway improved the glycolytic flux, thereby correcting some of the metabolic abnormalities of T2DM [70]. By increasing the NADH/NAD+ ratio, the polyol pathway inhibits glyceraldehyde 3-phosphate dehydrogenase, an enzyme of the glycolytic pathway. Vice versa, inhibition of AR corrects this effect, thereby increasing glycolytic flux [70].

Interestingly, overexpression of AR in mouse hearts impaired metabolic recovery after ischemia and reperfusion. Left ventricular developed pressure recovery was significantly reduced, and ATP content was lower compared to wild-type mouse hearts, showing that AR may play a key role in ischemic injury and metabolic recovery after ischemia [71]. The question arises as to whether these findings are applicable to hereditary/sporadic higher AR levels in clinical populations. Genetic mapping of the AR protein among Japanese individuals with T2DM showed that it may serve as a genetic marker of susceptibility toward cerebrovascular strokes [72]. Patients treated with an AR inhibitor showed an increase in left ventricular function and left ventricular stroke volume [73].

The novel AR inhibitor Caficrestat showed promising results in the treatment of patients with COVID-19 and co-morbid diabetes mellitus and heart disease: In these patients, Caficrestat reduced mortality and the length of hospitalization, although the results did not reach significance [74].

Despite these promising findings, clinical translation of AR inhibitors has been limited. AR inhibitors have been withdrawn or have not been approved (e.g., in the US) due to safety concerns. One major issue is their poor selectivity: While they inhibit AR, they may interfere with detoxification pathways for harmful metabolites produced during oxidative stress and can also affect anti-inflammatory responses. These off-target effects have raised concerns about their long-term safety and efficacy in patients [75].

In brief, AR inhibitors may still hold significant therapeutic potential for patients with diabetic and cardiovascular complications, but further refinement of their pharmacological profiles and safety is essential for broader clinical use.

### 3.3. Study Limitations

H9c2 cardiomyoblasts are a proliferating cell line, in contrast to the non-proliferating nature of primary cardiomyocytes. Mammalian neonatal cardiomyocytes are more dependent on glucose and possess a higher glycolytic activity compared to adult cardiomyocytes, which almost exclusively depend on oxidative metabolism, mainly through the utilization of free fatty acids [76,77,78,79,80]. Therefore, neonatal hearts are rather resistant to hypoxia, and a substantially longer time of hypoxia was needed to show sufficient injury compared to an in vivo model. Additionally, our model represents a homogeneous population of cardiac cells. In other words, surrounding cell types (e.g., cardiac fibroblasts, myocytes, endothelial cells, and vascular smooth muscle cells) and their interactions are missing.

Moreover, H9c2 cells were cultured in Dulbecco’s Modified Eagle Medium (DMEM), which already contains 25 mM glucose as a standard component. To investigate hyperglycemic stress, additional glucose was added. Although these elevated levels do not reflect physiological states, they were used to induce dose-dependent metabolic stress and assess the protective effects of Epalrestat.

It should be noted that cells were exposed to these conditions for only 24 h, whereas T2DM is a complex, multifactorial disease that develops gradually over extended periods and involves diverse pathophysiological processes, such as insulin resistance, metabolic shifts, lipotoxicity, hypertrophy, and altered cardiac function [81].

We did not explore varying durations of reoxygenation; based on previous work, we have chosen a duration of 2 h for reoxygenation [82].

While our study used Fluo-4 to measure total intracellular calcium levels, we did not assess calcium fluxes or pharmacologically dissect the specific sources of calcium. Additionally, membrane potential was not evaluated, which limits our mechanistic understanding of mitochondrial involvement.

Furthermore, intracellular AR activity was not measured in this study. The expression of AR of the H9c2 cardiomyoblasts in this study might not be at levels similar to those in humans. Like other cell models, this may limit the degree of translatability of our in vitro results to human physiology [83].

We acknowledge that relying solely on LDH release limits the depth of insight into the mechanisms of cell injury and death. In future studies, we aim to incorporate complementary assays to more comprehensively assess cell viability, apoptosis, and necrosis. Additionally, while this study did not directly measure AR activity or expression, future investigations could employ spectrophotometric enzyme activity assays or Western blot analysis to evaluate the inhibitory effect of Epalrestat at the molecular level.

At the time of the study, other AR inhibitors, such as Fidarestat, were not commercially available and could therefore not be included as a comparator. However, given the recent commercial availability of other AR inhibitors, future studies should consider directly comparing different AR inhibitors to further elucidate their respective protective effects.

Finally, the limited overall number of observations stemming from the extended duration of the experiments contributes to reduced statistical power and the potential for a type II error.

## 4. Materials and Methods

### 4.1. Cell Culture

H9c2(2-1) embryonic rat cardiomyoblasts, as well as the culture media, were obtained from ATCC (Manassas, VA, USA). H9c2(2-1) cells, derived from embryonic rat ventricular tissue, are widely used as an in vitro model to study cardiac metabolism, apoptosis, and oxidative stress under pathophysiological conditions such as hyperglycemia and IR. Due to their cardiomyocyte-like properties and reproducible stress responses, they are considered a suitable model for investigating cardioprotective agents, including in hypoxia/reoxygenation and high-glucose environments. We chose H9c2 cardiomyoblasts because they are energetically more similar to primary cardiomyocytes than other commonly used cell lines, like atrial HL-1 mouse cells [76,77]. The cells were cultured in Dulbecco’s modified Eagle’s medium (DMEM), containing 25 mM glucose, supplemented with 10% fetal bovine serum (FBS) and 5% Penicillin/Streptomycin (Gibco by Thermo Fisher Scientific, Waltham, MA, USA). The cells were incubated at a standard cell culture environment of humidified 21% O_2_, 74% N_2_, 5% CO_2_ at 37 °C, and sub-cultured when at 70% confluence.

For all experiments, cardiomyocytes between passages 6 and 25 were plated at a seeding density of 30,000 cells/cm^2^ (10,000 cells/well; surface area of 1 well in a 96 well plate is 0.32 cm^2^) in clear-walled, clear-bottom, 96-well plates. All experiments were performed 24 h after plating.

### 4.2. Optimization of Hypoxia/Reoxygenation Conditions for Confluent H9c2 Cardiomyocytes

In in vitro models, hypoxia/reoxygenation (HR) is commonly used to simulate IR. Hypoxia can be induced by replacing the cell culture atmosphere with a hypoxic gas mixture; concomitantly, the cell culture media are changed to serum- and glucose-free media. In the reoxygenation period, normal cell culture conditions are restored.

To optimize the HR conditions for H9c2 cells, a series of experiments were performed. The amount of damage to the H9c2 cells is critical for a valuable injury model. On one hand, injury needs to be demonstrated, and it needs to be replicable. On the other hand, excessive damage would not allow for the examination of the protective actions of a given agent (e.g., the AR inhibitor Epalrestat) and would not allow for the inspection of additional damage from high glucose concentrations, as the maximum injury level would have already been reached. To determine the amount of time the cells should be exposed to hypoxia in further experiments, confluent H9c2 cells were exposed to 3, 6, 12, 24, and 48 h of hypoxic conditions (simulated ischemia; glucose- and serum-free media; 0.01% O_2_, 5% CO_2_, 94.99% N_2_; 37 °C; hypoxia incubator chamber) and 2 h of reoxygenation (simulated reperfusion; complete media; 21% O_2_, 5% CO_2_ 74% N_2_; 37 °C). After the two-hour reoxygenation period, the endpoints were assessed: Cell viability using the PrestoBlue^®^ cell reagent kit and the release of lactate dehydrogenase (LDH) were measured. PrestoBlue^®^ stains viable cells; the fluorescence of the viable cells can be quantified using a plate reader (fewer viable cells per well result in less fluorescence). LDH is an intracellular enzyme; if the cell membrane integrity is compromised, LDH leaks into the media. LDH in the media can be measured using an LDH assay kit and a plate reader (increasing fluorescence indicates a higher leak of LDH and cell damage). Twenty-four hours of hypoxia, although a notably longer duration compared to in vivo or ex vivo models, appeared to be the most suitable to induce sufficient injury for the in vitro model without injuring the cells too extensively (for detailed results, see Appendix B).

### 4.3. High Glucose Exposure and Hypoxia/Reoxygenation Injury Model

As previously presented [84], confluent cardiomyocytes were exposed to 25, 50, 75, 100, and 125 mM glucose concentrations or 25 mM glucose concentration with the addition of 25, 50, 75, and 100 mM sucrose concentrations (resulting in total molarities of 50, 75, 100, and 125 mM) for 24 h.

H9c2(2-1) cells were cultured in Dulbecco’s Modified Eagle Medium (DMEM), which contains 25 mM glucose as a standard component. This concentration is commonly used for H9c2 cells and serves as the baseline condition in this study. Attempts to reduce glucose concentrations to more normoglycemic levels (e.g., 5.6 mM) using glucose-free DMEM supplemented with “physiological glucose” concentrations resulted in impaired cell growth, reduced cell size, and loss of viability during extended culture, consistent with previous reports. To investigate the effects of hyperglycemic stress, additional glucose was added to achieve final concentrations of 50, 75, 100, and 125 mM. These concentrations were not intended to represent physiological states, but rather to induce dose-dependent metabolic stress for evaluating the cellular effects of elevated glucose and the potential protective effects of Epalrestat.

Mannitol is frequently used as an osmolarity control for high-glucose exposure models. However, it was shown to scavenge free radicals, lowering the concentration of ROS [85]. L-glucose, another non-metabolizable sugar, is a valid alternative but is less commonly available and more expensive. In this study, sucrose was chosen as the osmolarity control because it is a non-metabolizable disaccharide that does not enter mammalian cells, lacks antioxidant properties, is cost-effective, and is commonly used in in vitro models. Additionally, sucrose solutions exert osmotic pressure comparable to that of glucose solutions at equivalent concentrations, making sucrose a suitable control for assessing osmotic effects.

The cells were then subjected to either 24 h of hypoxic conditions (simulated ischemia; glucose/sucrose- and serum-free media; 0.01% O_2_, 5% CO_2_, 94.99% N_2_; 37 °C; hypoxia incubator chamber) or normoxic conditions (complete media with corresponding glucose or sucrose concentrations; 21% O_2_, 5% CO_2_ 74% N_2_; 37 °C). Cells in the hypoxia group were deprived of glucose, serum, and oxygen to mimic ischemia during which transport of oxygen and nutrients to the cells are reduced due to inadequate blood supply. This was followed by a two-hour reoxygenation period (simulated reperfusion) in normoxic conditions, during which the regular media was reintroduced (also containing the corresponding glucose or sucrose concentrations). After 50 h, the endpoints were assessed. For the endpoint assessment, cell viability, cell damage (LDH release), cell membrane damage (FM1-43 incorporation into cell membrane), intracellular calcium accumulation (Fluo-4 assay kit), and ROS formation, specifically, superoxide anion formation (dihydroethidium [DHE] fluorescence) were measured.

### 4.4. Aldose Reductase Inhibitor—Epalrestat

The AR inhibitor Epalrestat was purchased from Sigma-Aldrich, St. Louis, MO, USA. A stock solution was prepared using deionized water. To test for potential cytotoxic effects of Epalrestat, three wells, respectively, were exposed to final concentrations of 0, 1, 5, 10, and 20 µM Epalrestat in 100 µL media per well. LDH release was measured after 24 h. There was no significant difference in damage within the range of these concentrations. As previously presented [86], confluent cardiomyocytes were exposed to 25, 50, and 100 mM glucose concentrations or 25 mM glucose concentrations with the addition of 25 or 75 mM sucrose concentrations (resulting in total molarities of 50 and 100 mM) for 24 h. The AR inhibitor was added when the cells were exposed to glucose or sucrose concentrations, resulting in final concentrations of 1 or 10 µM in 100 µL media per well. A group of control wells was not treated with Epalrestat. Four identical 96-well plates were prepared for each experiment. Two plates received different glucose concentrations (25, 50, and 100 mM), and two plates were used as osmolarity control. These plates received sucrose. Each well of these plates contained 25 mM glucose with additional sucrose, resulting in final molarities of 25 mM glucose + 25 mM sucrose = 50 mM and 25 mM glucose + 75 mM sucrose = 100 mM. After the two-hour reoxygenation period, cell injury was quantified via the colorimetric measurement of LDH in the media.

### 4.5. Endpoint Assessments

#### 4.5.1. Lactate Dehydrogenase Release

Cell injury was quantified by the colorimetric measurement of the LDH enzyme using the CytoTox 96^®^ Non-Radioactive Cytotoxicity assay kit (Promega, Madison, WI, USA). LDH is an intracellular enzyme. It leaks into the cell culture media when the cell membrane is damaged, which occurs when cells undergo apoptosis, necrosis, or other forms of cell damage [87]. At the end of the two-hour reoxygenation period, 50 µL of media from each well were transferred to a corresponding well of a new 96-well plate and mixed with 50 µL of prepared LDH reaction mixture. After 30 min of incubation at room temperature, the reactions were stopped by the addition of a 50 µL stop solution (which halts the reaction of tetrazolium salt to the formazan product). The absorbance of each well was measured from above at 490 nm using the plate reader (Synergy H1 Microplate Reader, BioTek Instruments, Inc., Winooski, VT, USA).

#### 4.5.2. FM1-43 Incorporation into the Cellular Membrane

As a measure of cell membrane damage, the membrane impermeant styryl dye FM1-43 (Molecular Probes, Inc., Eugene, OR, USA) was used. FM1-43 remains extracellular unless damage to the membrane allows it to become incorporated into the lipid bilayer of the cell membrane, where it fluoresces [82,88]. FM1-43 was added to each well at the beginning of the reoxygenation period with a final concentration of 10 µM per well. At the end of the 2-h reoxygenation period, the cells were washed with 1X phosphate-buffered saline (PBS); then, 100 µL of regular media was added back to the wells. The fluorescence was measured at excitation (Ex) of 488 nm and emission (Em) of 568 nm from below using the plate reader.

#### 4.5.3. Cell Viability and Cell Toxicity

Cell viability was assessed using the PrestoBlue^®^ cell reagent (Invitrogen by ThermoFisher Scientific, Waltham, MA, USA). After the two-hour reoxygenation period, the media was discarded, and 95 µL of fresh media and 5 µL of PrestoBlue^®^ cell reagent were added to each well. The cells were then incubated for 45 min at 37 °C in a humidified incubator. Using a plate reader, the fluorescence of each well was measured at Ex = 560 nm and Em = 590 nm read from below.

#### 4.5.4. Intracellular Calcium Accumulation

Intracellular calcium was assessed using the Fluo-4 Direct Calcium assay kit (Molecular Probes, Inc., Eugene, OR, USA). Fluo-4 is a fluorescent in-cell calcium indicator. At the start of the 2-h reoxygenation period, cells were loaded with 100 µL of the prepared 2X Fluo-4 direct-loading solution and 100 µL of regular media. After two hours, cells were washed with 1X PBS solution, and 100 µL of media was added back to the wells. The fluorescence of each well was read from below at Ex = 494 nm and Em = 576 nm using the plate reader.

#### 4.5.5. Reactive Oxygen Species Formation

Dihydroethidium (DHE) (Invitrogen by ThermoFisher Scientific, Waltham, MA, USA) is a superoxide indicator that exhibits blue fluorescence in the cytosol until oxidized, where it intercalates within the cell’s DNA, staining its nucleus a bright fluorescent red [89]. A 0.5 mM DHE stock solution was prepared using Milli-Q pure H_2_O for dilution. After the two-hour reoxygenation period, 2 µL of the 0.5 mM DHE stock solution was added to each well, resulting in a final concentration of 10 µM per well. Cells were incubated at 37 °C for 30 min. The fluorescence of each well was read from the below at Ex = 535 nm and Em = 595 nm using the plate reader.

### 4.6. Statistics

All data are expressed as percentages and normalized to 25 mM glucose after control/normoxic conditions (CN 25 mM). In every experiment and for each endpoint assay, the absolute values were normalized to the values of CN 25 mM (without any treatments per experiment). Thus, CN 25 mM was set to 100% within one experiment per endpoint. All data were tested for normal distribution using the Shapiro–Wilk test and for equal variance using the Brown–Forsythe and Welch test. If all tests were passed (*p* > 0.05), parametric testing was performed via a one-way analysis of variance (ANOVA). A Tukey’s range test was applied to find statistical differences among the groups. If one or all tests failed (*p* < 0.05), nonparametric testing was performed using the Kruskal–Wallis one-way ANOVA test, followed by Dunn’s method for all pairwise multiple comparison as a post-hoc test. Differences were considered statistically significant at *p* < 0.05 (two-tailed). Data are graphically presented as boxplots. Means are shown as “+”; boxes represent the 25th and 75th percentiles, and whiskers represent minimum to maximum. All data were analyzed using GraphPad Prism 10 (Version 10.1.0. GraphPad Software, San Diego, CA, USA).

## 5. Conclusions

Our results demonstrate that prior exposure of cardiomyocytes to high glucose concentrations sensitizes them to hypoxia and reoxygenation-induced injury. While normoxic cells maintained under high glucose conditions showed relatively preserved function, the combination of hyperglycemia and subsequent HR significantly exacerbated cell damage. These results underscore the role of hyperglycemia as a critical compounding factor that increases the vulnerability of cardiomyocytes to ischemic stress.

Moreover, with increasing glucose concentrations, a significant increase in the generation of superoxide anions was measured, even under normoxic conditions, suggesting that the formation of ROS may play a dominant role in the injury process. Adding the AR inhibitor Epalrestat during high glucose exposure attenuated cellular injury after HR. By inhibiting excessive glucose flux through the polyol pathway, Epalrestat appears to protect cardiomyocytes from baseline vulnerability to oxidative stress as well as the creation of additional oxidative stress and inflammatory responses during IR.

## Figures and Tables

**Figure A1:**
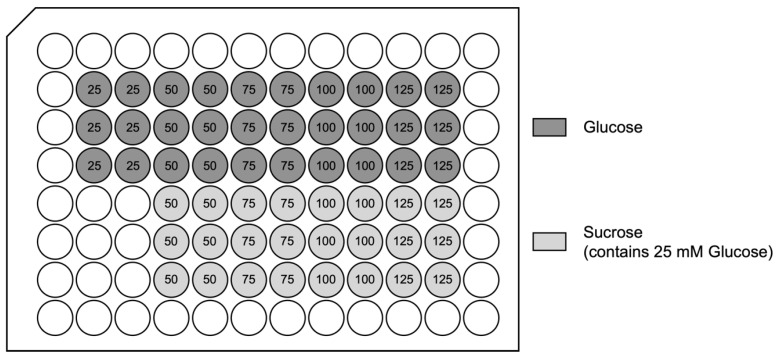
96-well plate set-up: high glucose exposure hypoxia/reoxygenation injury model: The numbers show total molarities in mM. For each experiment, two identical 96-well plates were prepared. One was exposed to hypoxic conditions, while the other plate underwent normoxic conditions.

**Figure A2:**
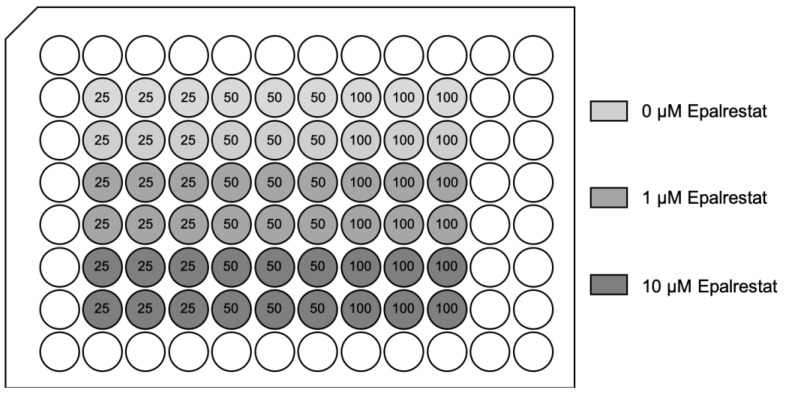
96-well plate set-up: treatment with aldose reductase inhibitor (Epalrestat): Four identical plates were seeded for each experiment. Two glucose plates and two sucrose plates, where one plate underwent hypoxia/reoxygenation, while the other plate underwent normoxic conditions, respectively. The numbers show the final molarities in mM. For the osmolarity control sucrose plates, each well contained 25 mM glucose and additional sucrose, resulting in the final molarities shown in the 96-well plate set-up. The Epalrestat concentrations are final concentrations in 100 µL media per well.

**Figure A3:**
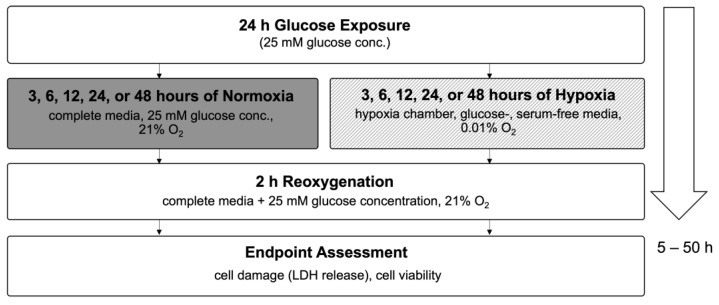
Flow chart of experimental timeline of varying hypoxia exposure times. Abbreviations: LDH, lactate dehydrogenase.

**Figure A4:**
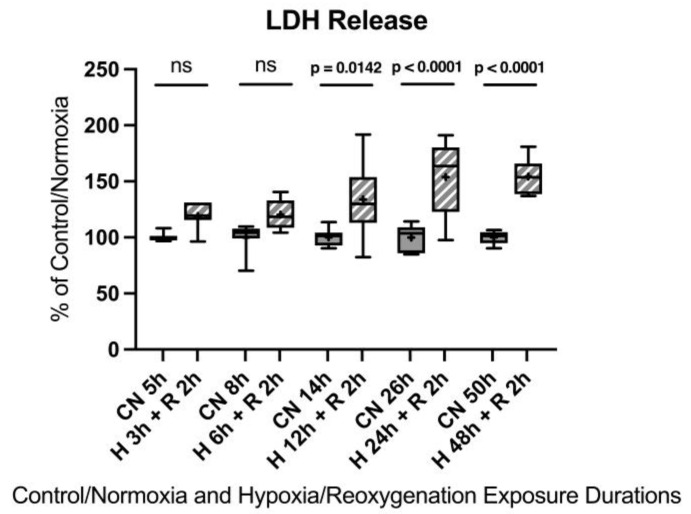
Hypoxia/reoxygenation titration model: Lactate dehydrogenase (LDH) release after 3, 6, 12, 24, or 48 h of hypoxia and 2 h of reoxygenation. Abbreviations: CN: control/normoxia; H: hypoxia; R: reoxygenation; n = 6 to 8 per group. All data were tested for normal distribution using the Shapiro–Wilk test and for equal variance using the Brown–Forsythe and Welch test. If all tests were passed (*p* > 0.05), parametric testing was performed via one-way analysis of variance (ANOVA). The Tukey’s range test was applied to find statistical differences among the groups. If one or all tests failed (*p* < 0.05), nonparametric testing was performed using the Kruskal–Wallis one-way ANOVA test, followed by Dunn’s method for all pairwise multiple comparison as a post hoc test. Differences were considered statistically significant at *p* < 0.05 (two-tailed). Data are graphically presented as boxplots. Means are shown as “+”; boxes represent the 25th and 75th percentiles, and whiskers represent minimum to maximum. Symbols: ns = not significant, *p* < 0.05 H 12 h + R 2 h vs. CN 14 h, *p* < 0.0001 H 24 h + R 2 h vs. CN 26 h, *p* < 0.0001 H 48 h + R 2 h vs. CN 50 h.

**Figure A5:**
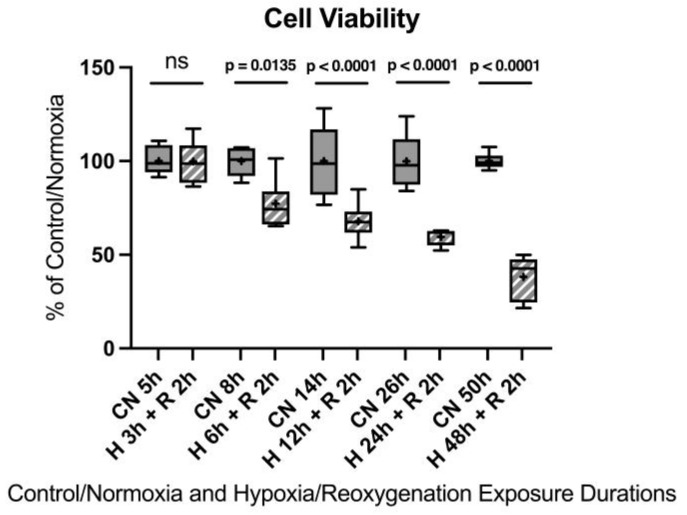
Hypoxia/reoxygenation titration model: Cell viability after 3, 6, 12, 24, or 48 h of hypoxia and 2 h of reoxygenation. Abbreviations: CN: control/normoxia; H: hypoxia; R: reoxygenation; n = 6 to 8 per group. All data were tested for normal distribution using the Shapiro–Wilk test and for equal variance using the Brown–Forsythe and Welch test. If all tests were passed (*p* > 0.05), parametric testing was performed via the one-way analysis of variance (ANOVA). The Tukey’s range test was applied to find statistical differences among the groups. If one or all tests failed (*p* < 0.05), nonparametric testing was performed using the Kruskal–Wallis one-way ANOVA test, followed by Dunn’s method for all pairwise multiple comparison as a post-hoc test. Differences were considered statistically significant at *p* < 0.05 (two-tailed). Data are graphically presented as boxplots. Means are shown as “+”; boxes represent the 25th and 75th percentiles, and whiskers represent minimum to maximum. Symbols: ns = not significant, *p* < 0.05 H 6 h + R 2 h vs. CN 5 h, *p* < 0.0001 HR 12 h + 2 h vs. CN 14 h, *p* < 0.0001 H 24 h + R 2 h vs. CN 26 h, *p* < 0.0001 H 48 h + R 2 h vs. CN 50 h.

## Data Availability

All data are available upon reasonable request and in accordance with funding guidelines.

## Abbreviations

The following abbreviations are used in this manuscript:

AR	Aldose reductase
CN	Control/Normoxia
CVDs	Cardiovascular diseases
DHE	Dihydroethidium
Em	Emission
Ex	Excitation
GSH	Reduced glutathione
GSSG	Oxidized glutathione
HR	Hypoxia/reoxygenation
IR	Ischemia/reperfusion
LDH	Lactate dehydrogenase
NAD^+^	Nicotinamide adenine dinucleotide
NADH	Nicotinamide adenine dinucleotide + Hydrogen
NADP^+^	Nicotinamide adenine dinucleotide phosphate
NADPH	Nicotinamide adenine dinucleotide phosphate + Hydrogen
PBS	Phosphate-buffered saline
ROS	Reactive oxygen species
T2DM	Type 2 diabetes mellitus

## Appendix A

**Table A1:** Laboratory equipment.

Name	Company	Catalog No.
−20 °C Freezer	Frigidaire	FFU17FHW4
Synergy H1 Hybrid Multi-Mode Reader	BioTek Instruments, Inc.	H1MF
Bio-Safety Cabinet	NuAire	NU425400
Hypoxia Incubator Chamber	Stemcell Technologies	27310
Centrifuge	Anstel Enterprises Inc.	4235
Cell Counter	Invitrogen	C10281
Cell Culture Incubator	NuAire	Nu-5500
Refrigerator (4 °C, Cell Culture Room)	Kelvinator	MRT18NREW1
UltraRocker™ Rocking Platform	Bio-Rad Laboratories, Inc.	1660719
Liquid Nitrogen Tank (Cell Storage)	Thermo Fisher Scientific	CY509109
Various Volume Pipets	Mettler-Toledo Rainin, LLC	17008708
Inverted Light Microscope	Nikon	TMS214343
4 °C Refrigerator	Frigidaire	FFU17G4JW25
Inverted Laboratory Microscope	Leica Microsystems CMS GmbH	DM IL LED
CCD Microscope Camera	Leica Microsystems CMS GmbH	DFC365 FX
Software platform for life science LAS X Life Science	Leica Microsystems CMS GmbH	Version 2.0

**Table A2:** Chemical substances, reagents, and assay kits.

Name	Company	Catalog No.
PrestoBlue^®^ Cell Reagent	Invitrogen by Thermo Fisher Scientific	A13262
CytoTox 96^®^ Non-Radioactive Cytotoxicity Assay Kit	Promega	G1780
Fluo-4 Direct™ Calcium Assay Kit	Invitrogen by Thermo Fisher Scientific	F10471
Dihydroethidium (Hydroethidine)	Invitrogen by Thermo Fisher Scientific	D11347
Membrane Impermeant Styryl Dye FM™1-43	Molecular Probes	T3163
Epalrestat	Merck by Sigma-Aldrich	SML0527
D-(+)-Glucose	Sigma-Aldrich	G8270
Sucrose	Sigma-Aldrich	S0389
Ethanol, 200 Proof	Thermo Fisher Scientific	BP2818
Sterile Water	Thermo Fisher Scientific	I9030
AquaGuard Water Bath Cleaner	Thermo Fisher Scientific	19161E
CO_2_ Cylinder for Cell Incubator	A-L Compressed Gases	UN1013
0.01% O_2_ Cylinder (Hypoxic Mixture)	A-L Compressed Gases	UN1956
Liquid Nitrogen Tank	A-L Compressed Gases	UN4052

**Table A3:** Expendables.

Name	Company	Catalog No.
96-well Clear Flat Bottom Polystyrene TC-treated Microplate, with Low Evaporation Lid, Sterile	Corning	1016742
Sterile Syringe Filter 0.22 µm	Fisherbrand	09720004
Cell Counting Chambers w/Trypan Blue Dye	Fisher	C10228
10 mL Pipette	CELLSTAR Serological Pipettes; Greiner Bio-One	607 180
50 mL Pipette	CELLSTAR Serological Pipettes; Greiner Bio-One	768 180
15 mL and 50 mL Conical Centrifuge Tubes	Thermo Fisher Scientific	22-171-716 and 14-432-22
Microcentrifuge Tubes (1.5 mL)	Research Products International	145515A
Reagent Reservoirs 25 mL, Sterile	Thermo Fisher Scientific	8096-11
Pipet Tips, Various Sizes to Fit Rainin Pipets	VWR	83009-688
Plastic Weigh Boats, Small	VWR	611-9171
Paper Towels	Thermo Fisher Scientific	06-666-114
Bench Protectors (Blue Pads)	VWR	82020-845
Delicate Task Wipes	KimTech	34155
Nitrile Gloves	B. Braun	9209817

**Table A4:** Cell culture.

Name	Company	Catalog No.
H9c2(2-1) clonal cell line derived from embryonic BD1X rat heart tissue	ATCC	CRL-1446
Dulbecco’s Modified Eagle’s Medium (DMEM)	ATCC	30-2002
Fetal Bovine Serum (FBS)	ATCC	30-2020
Penicillin-Streptomycin 10.000 U/mL	Gibco by Thermo Fisher Scientific	15140122
Dulbecco’s Modified Eagle’s Medium (DMEM), Glucose- and Serum Free, Glutamine Free, without Phenol Red	Gibco by Thermo Fisher Scientific	A1443001
Trypsin-EDTA (0.25%) Phenol Red (100 mL)	Gibco by Thermo Fisher Scientific	25200056
1X Phosphate-Buffered Saline (500 mL)	Thermo Fisher Scientific	J61196.AP
H9c2(2-1) clonal cell line derived from embryonic BD1X rat heart tissue	ATCC	CRL-1446
Dulbecco’s Modified Eagle’s Medium (DMEM)	ATCC	30-2002
Fetal Bovine Serum (FBS)	ATCC	30-2020
Penicillin-Streptomycin 10.000 U/mL	Gibco by Thermo Fisher Scientific	15140122
Dulbecco’s Modified Eagle’s Medium (DMEM), Glucose- and Serum Free, Glutamine Free, without Phenol Red	Gibco by Thermo Fisher Scientific	A1443001
Trypsin-EDTA (0.25%) Phenol Red (100 mL)	Gibco by Thermo Fisher Scientific	25200056
1X Phosphate-Buffered Saline (500 mL)	Thermo Fisher Scientific	J61196.AP

## Appendix B

### Appendix B.1. Optimization of Hypoxia Exposure Time

To optimize the HR protocol for H9c2 cardiomyocytes, cells were exposed to 3, 6, 12, 24, and 48 h of hypoxia, followed by two hours of reoxygenation in normoxic conditions or just normoxic conditions of the same duration. Cell damage was then evaluated using the LDH release assay kit and the PrestoBlue^®^ cell viability reagent assay kit.

### Appendix B.2. Cell Damage Assessed by Lactate Dehydrogenase Release

No significant differences were observed after 3 h and 6 h of hypoxia. However, 12 h of hypoxia resulted in a 33.9% increase in LDH release, 24 h showed a 53.9% increase, and 48 h led to a 54.2% increase.

### Appendix B.3. Cell Viability and Toxicity

After 3 h of hypoxia, no significant differences were observed. After 6 h of hypoxia, a 22.7% decrease in cell viability was detected compared to the control group. Twelve hours of hypoxia resulted in a 32.1% decrease in cell viability, 24 h showed a 40.4% decrease in cell viability, and 48 h led to a 61.8% decrease in cell viability. Twenty-four hours of hypoxia, although a notably longer duration compared to an in vivo model, appeared to be the most suitable to induce sufficient injury for the in vitro model without injuring the cells too extensively.
